# Bacterial Growth Modulatory Effects of Two Branched-Chain Hydroxy Acids and Their Production Level by Gut Microbiota

**DOI:** 10.4014/jmb.2404.04009

**Published:** 2024-05-10

**Authors:** Chan Hyuk Hwang, Su-Hyun Kim, Choong Hwan Lee

**Affiliations:** 1 Department of Bioscience and Biotechnology, Konkuk University, Seoul 05029, Republic of Korea; 2 MetaMass Corp., Seoul 05029, Republic of Korea

**Keywords:** Gut microbiota, branched-chain hydroxy acid, bacterial growth modulation, antibacterial effect, quantitation, *Lactobacillaceae*

## Abstract

Branched-chain hydroxy acids (BCHAs), produced by lactic acid bacteria, have recently been suggested as bioactive compounds contributing to the systemic metabolism and modulation of the gut microbiome. However, the relationship between BCHAs and gut microbiome remains unclear. In this study, we investigated the effects of BCHAs on the growth of seven different families in the gut microbiota. Based on in vitro screening, both 2-hydroxyisovaleric acid (HIVA) and 2-hydroxyisocaproic acid (HICA) stimulated the growth of *Lactobacillaceae* and *Bifidobacteriaceae*, with HIVA showing a significant growth promotion. Additionally, we observed not only the growth promotion of probiotic *Lactobacillaceae* strains but also growth inhibition of pathogenic *B. fragilis* in a dosedependent manner. The production of HIVA and HICA varied depending on the family of the gut microbiota and was relatively high in case of *Lactobacillaceae* and *Lachnosporaceae*. Furthermore, HIVA and HICA production by each strain positively correlated with their growth variation. These results demonstrated gut microbiota-derived BCHAs as active metabolites that have bacterial growth modulatory effects. We suggest that BCHAs can be utilized as active metabolites, potentially contributing to the treatment of diseases associated with gut dysbiosis.

## Introduction

The human gut microbiome, composed of diverse lineages of microorganisms, contributes to the metabolic health of the host [1]. Various phyla, including Firmicutes, Proteobacteria, Bacteroidetes, and Actinomycetota, dominate the human gut microbiome [2-4]. Recently, the importance of maintaining a balanced gut microbiota has been increasingly emphasized. An imbalance in the gut microbiome, known as dysbiosis, can cause various metabolic diseases such as obesity, type 2 diabetes, non-alcoholic fatty liver disease, and cardiometabolic disease [5-8]. Considerable research has been conducted on the modulation of bacteria to balance the gut microbiome. Improvement of the intestinal environment provides health benefits to the host [9, 10]. The gut microbiome can be improved by promoting the growth of probiotics through prebiotics and inhibiting the growth of pathogens through antibiotics [11-14]. Various metabolites such as dietary fibers and polyphenols have been proposed as prebiotics [15-17]. Recent studies have reported that microorganism-derived metabolites can modulate the gut microbiome [18-21]. For example, short-chain fatty acids produced by the gut microorganisms not only improve the host’s intestinal environment and affect the gut-brain axis [22], but also change the composition of the gut microbiota [23]. As an increase in probiotics is important for the host’s health [24-26], the growth modulation of the gut microbiome by these metabolites must be investigated.

Branched-chain hydroxy acids (BCHAs) are metabolites derived from branched-chain amino acids (BCAAs) and produced by lactic acid bacteria (LAB) [27]. Valine, leucine, and isoleucine are converted to 2-hydroxyisovaleric acid (HIVA), 2-hydroxyisocaproic acid (HICA), and 2-hydroxy-3-methylvaleric acid (HMVA), respectively. Intestinal cell proliferation and antifungal activity have been reported for HIVA [28, 29], and alleviation of muscle atrophy and antifungal and antibacterial activities have been reported for HICA [29-32]. A recent study has shown that BCHAs regulate the action of insulin on glucose metabolism and alter the composition of the gut microbiota [33]. However, the growth-modulatory effects of BCHAs on the gut microbiota have not been evaluated.

In this study, BCHAs production level of the gut microbiota was determined using GC-TOF-MS. Then, the growth-modulatory effects of BCHAs on the gut microbiota were investigated through 96-well plate-based in vitro screening. Additionally, the growth modulation by BCHAs on the gut microbiota were then validated. Based on this, we sought to reveal the interaction between BCHAs and the gut microbiota.

## Materials and Methods

### Chemicals and Reagents

High performance liquid chromatography (HPLC) grade methanol (MeOH) and water were purchased from Thermo Fisher Scientific (USA). All analytical reagents used in this study were obtained from Sigma Chemical Co.(USA). HIVA (CAS No. 17407-56-6), HICA (CAS No. 498-36-2), N-methyl-N-(trimethylsilyl) trifluoroacetamide (MSTFA), methoxyamine hydrochloride, and pyridine were purchased from Sigma (USA). The brain heart infusion (BHI) broth was purchased from Kisan Biotech (Republic of Korea).

### Culture Conditions for Gut Microbial Strains

**Fig. 1:**
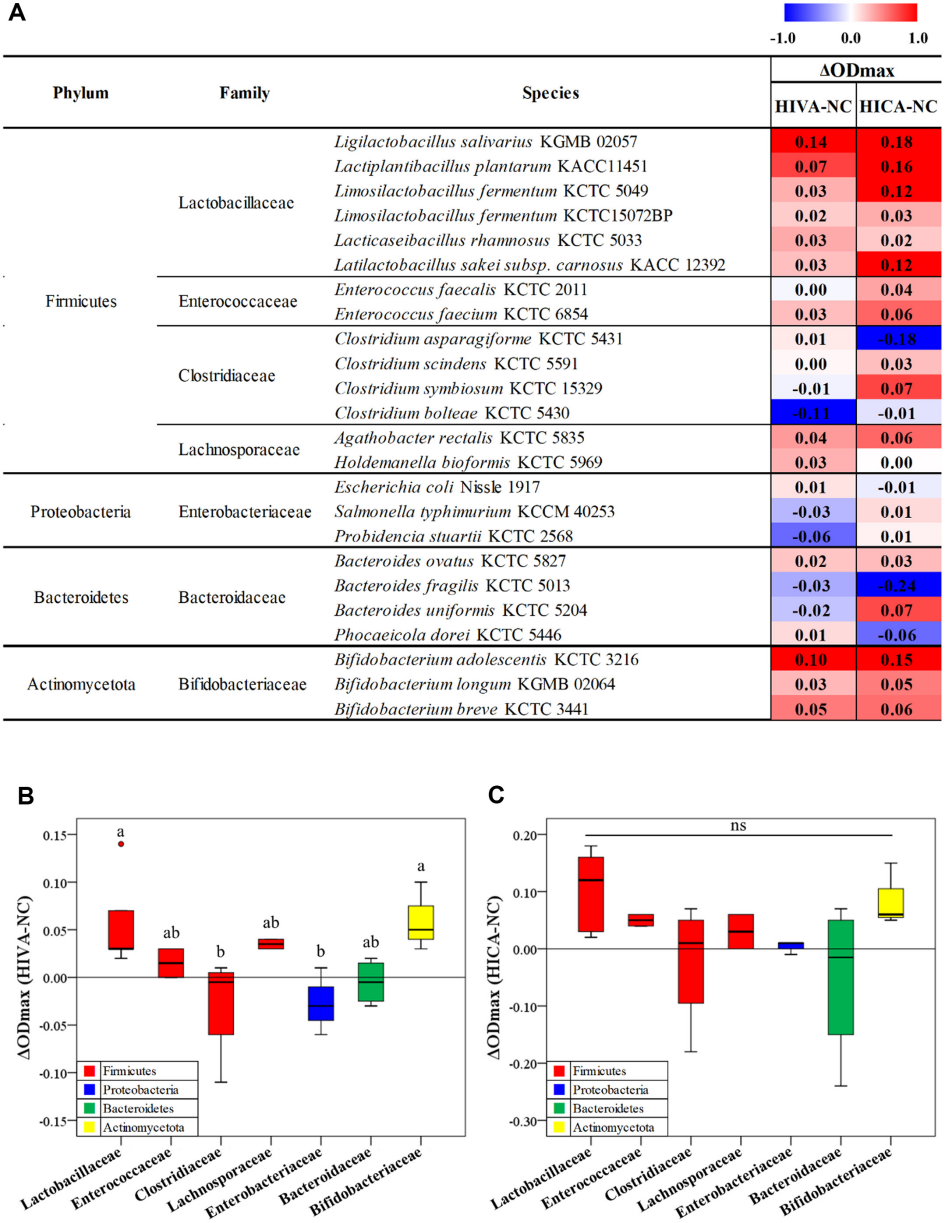
Bacterial growth modulation by HIVA and HICA on the gut microbiota at the (**A**) species level and (**B, C**) family level. (**A**) The heat map represents the differences in the maximal optical density at the species level compared with negative control (**NC**) by HIVA and HICA treatment. The box plots represent the differences by (**B**) HIVA and (**C**) HICA at family level. All values are expressed as the average of three biological replicates. Different letters above the box plots in (**C**) and (**C**) indicate significant differences according to Duncan’s multiple range test (*p* < 0.05).

The gut microbial strains used in this study are listed in Fig. 1A. We selected 24 different microbial strains across major phyla (Actinobacteria, Firmicutes, Bacteroidetes, and Proteobacteria) prevalent in the human gastrointestinal tract. All bacterial strains were cultured anaerobically on BHI agar supplemented with 10% defibrinated horse blood (Kisan Biotech). The corresponding submerged cultures were grown in BHI medium supplemented with 0.05% cysteine. The strains were cultured under anaerobic condition at 37°C for 24 h. A flexible anaerobic chamber (Coy Laboratory Products, USA) containing 10% CO_2_, 5% H_2_, and 85% N_2_ was used for culture maintenance and all other microbiological experiments under anaerobic conditions.

### In vitro Screening for Growth Modulation of Gut Microbes by HIVA and HICA

Gut bacteria were precultured separately in BHI broth supplemented with 0.05% cysteine (5 ml) at 37°C for 24 h. Each preculture was then inoculated into 0.2 ml of fresh BHI broth containing HIVA and HICA (0.25 mg/ml) to OD600 = 0.01 in a 96-well plate in triplicate. BHI medium was used as a negative control. The 96-well plate was sealed with an adhesive tape to maintain anaerobic conditions inside the plate. The plates were incubated at 37°C for 24 h in a spectrophotometer, and optical density (OD) was recorded automatically every 15 min at 600 nm with shaking for 3 s prior to each reading using a microplate reader. Growth was normalized with the background reads for the media and subtracted from the reads of each incubated sample. Growth curves were constructed separately for each microbe by fitting the data to the Baranyi model using Microsoft Excel. The maximal optical density (ODmax) was derived directly from the model.

### Validation for Growth Modulation of Probiotics and Pathogens by HIVA and HICA

*Lactiplantibacillus plantarum* KACC 11451, *Limsilactobacillus fermentum* KCTC15072BP, *Ligilactobacillus salivarius* KGMB 02057, and *Bacteroides fragilis* KCTC 5013 were precultured separately in BHI broth supplemented with 0.05% cysteine (5 ml) at 37°C for 24 h. Each preculture was then inoculated into 0.2 ml BHI broth containing HIVA, HICA (0.125–4.0 mg/ml), and inulin (0.25 mg/ml) as a positive control to OD600 = 0.01 in a 96-well plate in triplicate. BHI medium was used as a negative control. Growth was measured using the same method as described earlier.

### Preparation of Samples for Quantitation of BCHAs Produced by Gut Microbiota

Gut bacteria were precultured separately in BHI broth supplemented with 0.05% cysteine (5 ml) at 37°C for 24 h. Each preculture was then inoculated into 1.5 ml of fresh BHI broth at OD600 = 0.01 in a 96-deep well plate (Bioneer, Republic of Korea). The plates were incubated at 37°C in an anaerobic chamber (H_2_ 5%, CO_2_ 10%, and N_2_ 85%).

Each bacterial culture samples (800 μl) were collected 24 h after incubation at 37°C, centrifuged at 10,000 ×*g* for 10 min, and filtered using Millex GP 0.22-μm filter (Merck Millipore, USA) to separate the supernatant. The separated supernatant (200 μl) was added to 800 μl of 100% cold MeOH, vortexed for 30 s, and centrifuged at 24,249 ×*g* for 10 min at 4°C. The filtered supernatant was dried using a speed vacuum concentrator. For instrumental analysis, the dried samples were dissolved in 1 ml of 100% MeOH.

To prepare standard solutions, stock solutions of HICA and HIVA were serially diluted to concentrations of 0.004, 0.008, 0.016, 0.03, 0.06, 0.12, 0.25, 0.5, 0.1, and 0.2 μg/ml in 100% MeOH. 2-Chlorophenylalanine at a concentration of 10 μg/ml was used as an internal standard.

### Gas Chromatography – Time-of-Flight – Mass Spectrometry (GC-TOF-MS) Analysis

The dried samples were subjected to two derivatization steps prior to GC-TOF-MS analysis. First, oximation was performed by adding 50 μl of methoxyamine hydrochloride in pyridine (20 mg/ml) to the dried samples and incubating at 30°C and 300 rpm for 90 min. Next, silylation was performed by adding 50 μl of MSTFA to the reaction mixture, followed by incubation at 37°C and 300 rpm for 30 min.

All samples were filtered using Millex GP 0.22-μm filter prior to GC-TOF-MS analysis performed using Agilent 7890A GC system equipped with Agilent 7693 autosampler and Pegasus BT TOF-MS. An Rtx-5MS capillary column (30 m × 0.25 mm × 0.25 μm, Restek Corp., USA) was used, and the operational parameters were adapted from a study reported by Lee *et al* [34]. A total of 1 μl of derivatized samples was injected into GC-TOF-MS with a split ratio of 30:1. All analyses were performed in a random order to reduce bias.

### Statistical Analysis

The growth variation by BCHAs were calculated based on differences of maximal OD and their fold-change abundances were expressed using a heat map. Significant differences in bacterial growth and production of HIVA and HICA were tested using one-way ANOVA with Duncan’s multiple range test and an independent samples *t*-test using PASW Statistics 18 (SPSS Inc., USA). The Pearson’s correlation coefficient between the production and growth modulation of HIVA and HICA was calculated using PASW Statistics 18.

## Results

### Growth Modulatory Effects of BCHAs on the Gut Microbiota

To investigate the effects of HIVA and HICA on the intestinal environment, we evaluated the effects of HIVA and HICA on growth of the gut microbiota through 96-well plate-based in vitro screening. Each microbe was monocultured in broths treated with 0.25 mg/ml of each HIVA and HICA. To investigate the variation in bacterial population size, we compared the ODmax of each species with that of the negative control.

Despite belonging to the same family, the growth-modulatory effect differed between species (Fig. 1A). Among *Lactobacillaceae* and *Bifidobacteriaceae*, *L. salivarius* and *B. adolescentis* were the most stimulated by both HIVA and HICA treatments. In *Bacteroidaceae* family, the growth of *B. ovatus* was stimulated; however, the growth of *B. fragilis* was inhibited when treated with HICA compared with HIVA.

In some strains, the growth-modulatory effect differed depending on HIVA or HICA treatment. The growth of *S. typhimurium* and *P. stuartii* was inhibited by HIVA treatment, whereas no change was observed when treated with HICA. *B. fragilis* showed a greater decrease in growth by HICA treatment than that by HIVA treatment. In *B. uniformis*, HIVA inhibited growth, whereas HICA promoted growth. These results confirmed that the growth modulatory effects of HIVA and HICA depend on the bacterial species.

Notably, HIVA significantly stimulated the growth of *Lactobacillaceae* and *Bifidobacteriaceae* but significantly suppressed the growth of *Clostridiaceae* and *Enterobacteriaceae* compared with that of the negative control (Fig. 1B). HICA also stimulated the growth of *Lactobacillaceae* and *Bifidobacteriaceae*; however, the differences were not statistically significant (Fig. 1C). *Clostridiaceae* and *Bacteroidaceae* showed different trends between species in both HIVA and HICA treatment groups.

### Validation for the Growth Modulatory Effects of BCHAs on Probiotics and Pathogens

Based on the results of in vitro assay, HIVA and HICA were validated to investigate the effects of the two metabolites on probiotics and pathogens. Among *Lactobacillaceae*, which are mainly used as probiotics, *L. plantarum* KACC 11451, *L. fermentum* KCTC15027BP, *L. salivarius* KGMB 02057 that showed increased growth due to treatment with two substances were selected [36-38]. On the other hand, *B. fragilis*, known as pathogen, whose growth was reduced by treatment with two substances was selected and validated [35].

**Fig. 2:**
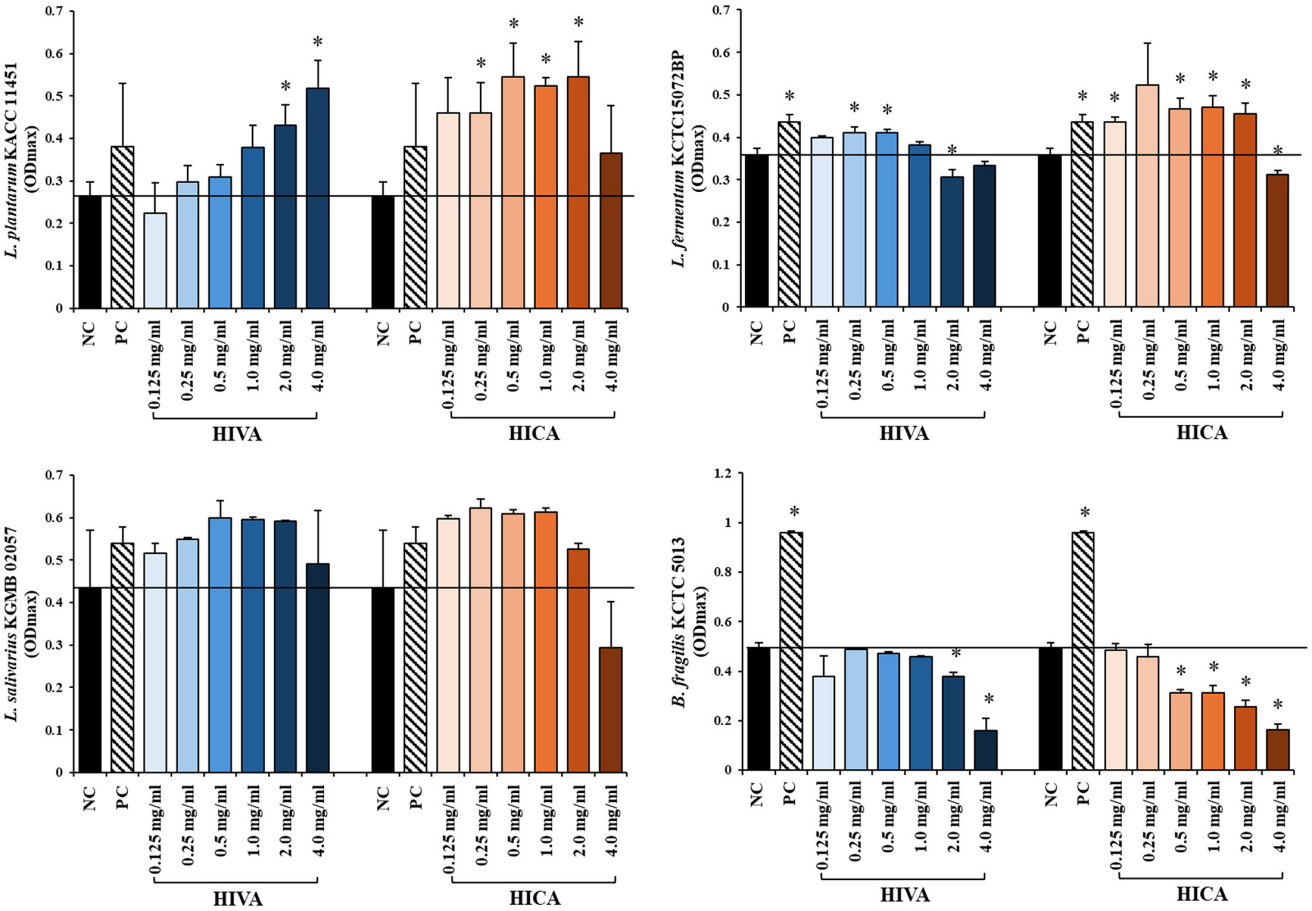
Bacterial growth modulation by HIVA and HICA at various concentrations on the growth of probiotics and pathogens. BHI broth was used as a negative control (**NC**) and inulin 0.25 mg/ml as a positive control (**PC**). (**A**) *L. plantarum* KACC 11451 (**B**) *L. fermentum* KCTC15072BP (**C**) *L. salivarius* KGMB 02057 (**D**) *B. fragilis* KCTC 5013. All values are expressed as the average of three biological replicates with standard deviation. The significance was determined using independent sample *t*-test (*p < 0.05).

Among all tested strains, *L. plantarum* was the most stimulated by the treatment with HIVA and HICA. At the tested concentrations, both metabolites promoted the growth of *L. plantarum*. Compared with the positive control (PC), both metabolites showed higher growth promoting effects at 2.0–4.0 mg/ml and 0.125–2.0 mg/ml, respectively. HIVA showed significant effects within the concentration range 2.0–4.0 mg/ml, whereas HICA showed significant effects within the concentration range 0.25–2.0 mg/ml. Notably, the highest growth promotimg effect was achieved when HICA was added at a concentration of 2.0 mg/ml (Fig. 2A).

For *L. fermentum*, the growth was stimulated when the concentrations of HIVA and HICA were in the range of 0.125–1.0 and 0.125–2.0 mg/ml, respectively. Compared with PC, HICA showed higher growth promoting effects at 0.25–2.0 mg/ml. HIVA exhibited significant effects within the concentration range of 0.25–0.5 mg/ml, while HICA showed significant effects at concentrations of 0.125 and 0.5–2.0 mg/ml. However, the growth was notably inhibited at concentrations of 2.0–4.0 mg/ml for HIVA and 4.0 mg/ml for HICA (Fig. 2B).

The growth of *L. salivarius* was stimulated within the concentration range of 0.125–4.0 and 0.125–2.0 mg/ml for HIVA and HICA, respectively. The growth was higher than PC at concentration of both metabolites in range of 0.25–2.0 and 0.125–1.0 mg/ml, respectively (Fig. 2C).

In contrast, pathogenic *B. fragilis* KCTC 5013 showed no significant change by HIVA and HICA treatments at 0.125–1.0 and 0.125–0.25 mg/ml, respectively. However, with increase in the concentrations of HIVA and HICA to 2.0–4.0 and 0.5–4.0 mg/ml, respectively, the growth of *B. fragilis* significantly decreased (Fig. 2D).

### Quantitation of BCHAs Production by the Gut Microbiota

To investigate the production of BCHAs by the gut microbiota, we cultured each bacterial species in BHI supplemented with cysteine and analyzed the extracellular sample extracts using GC-TOF-MS.

**Fig. 3:**
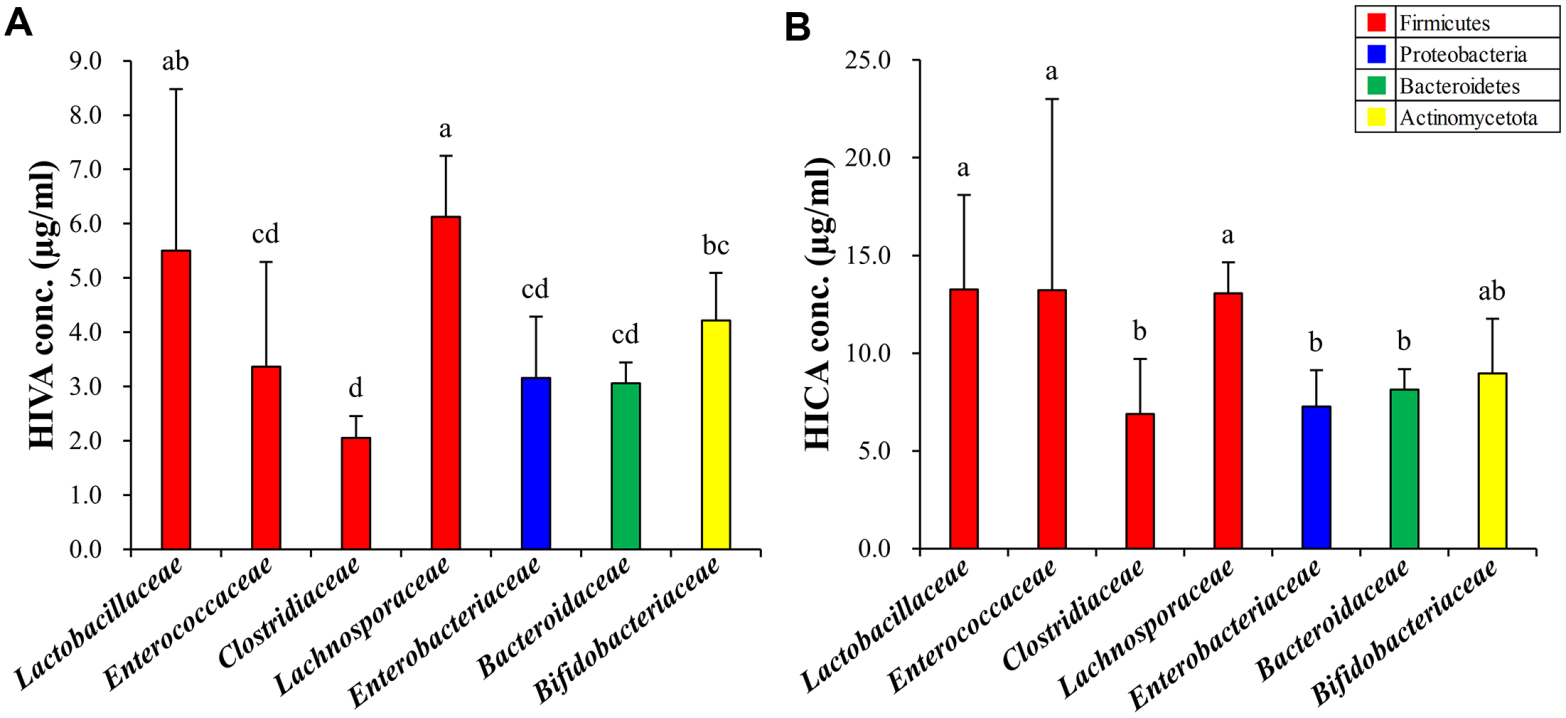
Production levels of (A) HIVA and (B) HICA by the gut microbiota at the family level. All values are expressed as the average of three biological replicates with standard deviation. The significance was determined using Duncan's multiple range test, and different letters above the bar graphs indicate significant differences (*p* < 0.05).

Both HIVA and HICA were detected in all gut microbiota and showed family-specific differences in their production (Fig. 3). Both HIVA and HICA production levels were significantly high in *Lactobacillaceae* and *Lachnosporaceae* and significantly low in *Clostridiaceae*, *Enterobacteriaceae*, and *Bacteroidaceae*. In *Enterococcaceae*, HICA production was significantly high, whereas HIVA production was significantly low. In case of *Bifidobacteriaceae*, HICA production was significantly high.

### Correlation between the Growth Variation and Production of BCHA

**Fig. 4:**
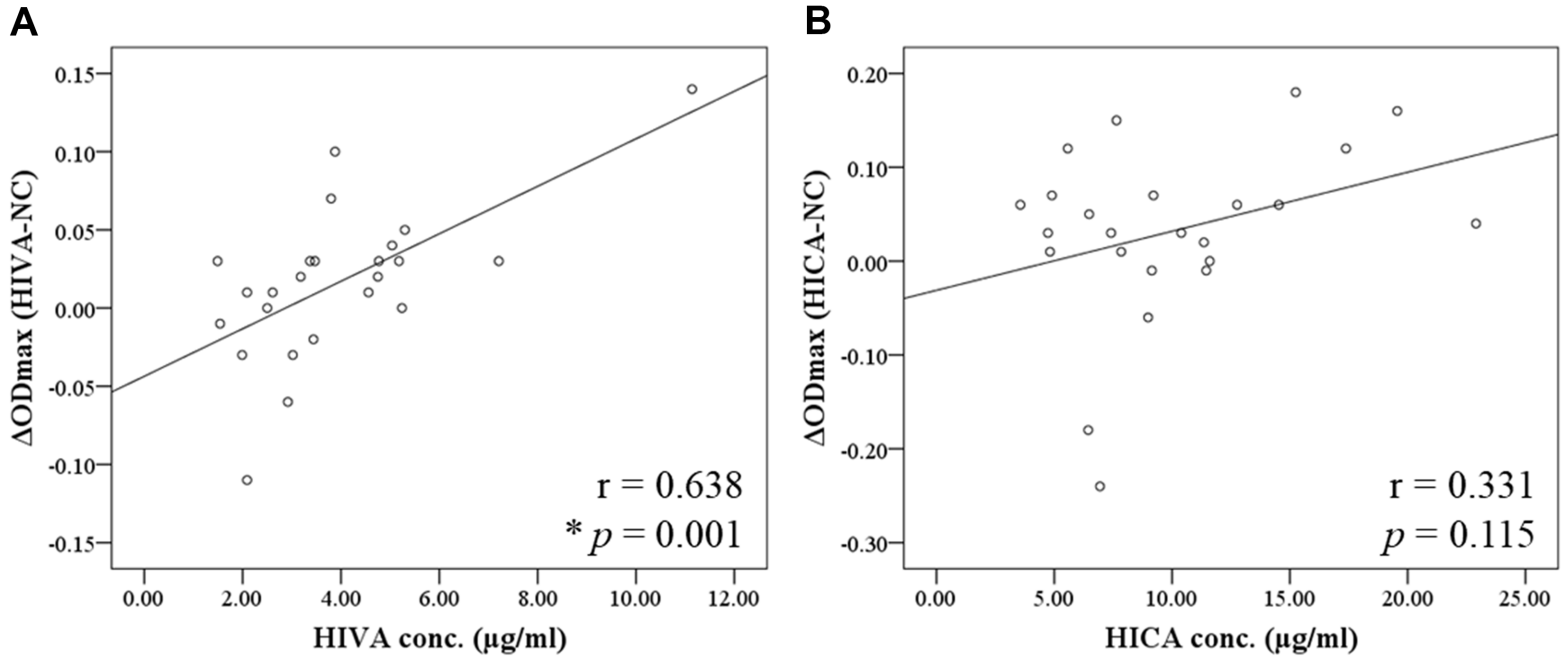
Correlation between the growth variation of the gut microbiota and their production of (**A**) HIVA and (**B**) HICA. Pearson correlation coefficient and *p*-values are shown. r, Pearson correlation coefficient; *p < 0.05.

To investigate the effects of HIVA and HICA on the intestinal environment, we conducted a statistical correlation analysis between the growth variation of the gut microbiota and their production of HIVA and HICA (Fig. 4). The correlation coefficient was larger for HIVA than that for HICA. HIVA showed significant strong positive correlation (r = 0.638, *p* = 0.001), whereas HICA showed a moderate positive correlation (r = 0.331, *p* = 0.115).

## Discussion

In this study, we investigated the growth modulation and production levels of BCHAs in gut microbiota to elucidate their impact on gut health as bioactive compounds. When BCHAs production by 24 selected strains in gut microbiota was examined, HIVA and HICA were detected at all strains, while HMVA was identified only in some strains. Regarding activities in modulating microbial growth, HIVA and HICA are known for anti-microbial and antifungal activities, but HMVA remains uncharacterized [29, 31, 32]. Therefore, we conducted screening and quantitation analysis of production focusing on HIVA and HICA, which have the potential to affect the growth of gut microbiota.

This study suggests novel findings regarding the effects of HIVA and HICA on the growth of gut microbiota. The effects of HIVA and HICA treatments varied depending on the bacterial family. Interestingly, treatment with HIVA or HICA increased the growth of *Lactobacillaceae* and *Bifidobacteriaceae*, which are the major probiotic bacteria that regulate the luminal pH, strengthen the intestinal barrier, secrete antibacterial peptides, and alter the composition of the gut microbiota [35, 36]. As probiotics, *Lactobacillaceae* and *Bifidobacteriaceae* play major roles through various metabolites derived from the intestinal microorganisms, and exhibit properties that improve the host’s health, including alleviating chronic diseases and stimulating the immune system [37-40]. Additionally, increased abundance of *Lactobacillaceae* and *Bifidobacteriaceae* in the intestinal environment has been reported to provide benefits to the patients with end stage renal disease[41]. Therefore, we confirmed that HIVA and HICA increased the abundance of the probiotic *Lactobacillaceae* and *Bifidobacteriaceae*, indicating that they are bioactive metabolites can improve the growth of probiotics.

In contrast, the growth of *Enterobacteriaceae* was inhibited by HIVA treatment. In a mouse model of inflammatory bowel disease, dysbiosis of the microbiota and increase in the abundance of *Enterobacteriaceae* was confirmed in response to inflammation [42]. Additionally, an increase in *Enterobacteriaceae* was associated with chronic ulcerative colitis, showing a positive correlation between this group of bacteria and severe disease stage [43]. Therefore, we suggest that HIVA may contribute to improving the health of the host by inhibiting the growth of pathogens.

In this study, HIVA showed better growth inhibition than HICA against gram-negative bacteria, including *S. typhimurium* KCCM 40253 and *P. stuartii* KCTC 2568. LAB have been reported to inhibit the growth of *S. typhimurium* [44]. HIVA produced by LAB might have contributed to these inhibitory effects. In the case of HICA, it is known to cause cell death through cell membrane penetration in some gram-negative bacteria [32]; however, the antibacterial activity of HIVA was unknown. Accordingly, we present HIVA as a metabolite with antibacterial activity.

Additionally, treatment with HIVA and HICA increased the growth of probiotic *Lactobacillaceae* species and suppressed the growth of *B. fragilis* in a concentration-dependent manner. In addition, *L. plantarum* regulates the production of inflammatory cytokines, including interleukin (IL)-1b, IL-6, IL-10, IL-12, and interferon-gamma, thereby modulating the balance of T cells crucial for immunity and consequently preventing IBD[45]. Colonization with *B. fragilis* has been shown to impair glucose tolerance and reduce insulin sensitivity [46]. Therefore, a reduction in *B. fragilis* may affect glucose metabolism. In this study, we confirmed that 2.0 mg/ml of HIVA and HICA stimulated the growth of probiotics, whereas the same concentration inhibited the growth of pathogens. This concentration is approximately 100 folds higher than the concentration contained in yogurt, which has shown physiological and gut microbiome-regulating activities [33]. These data suggest that HIVA and HICA can improve the host health by stimulating probiotics and inhibiting pathogens.

*L. plantarum* with increased growth produces 2-hydroxy acids, including BCHAs, which influence various physiological functions, including antibacterial, antioxidant, and immunomodulatory activities [47]. However, *B. fragilis* with suppressed growth has not yet been reported about BCHAs production. Thus, we analyzed the production pattern of BCHAs by the gut microbiota to investigate the correlation between BCHAs production and their growth modulatory effects. We investigated BCHAs production in four phyla of the gut microbiota using GC-TOF-MS. Among them, *Lactobacillaceae* produced both substances at relatively high levels compared to other family groups [48, 49]. We observed that the average HIVA and HICA production by *Lactobacillaceae* was 5.50 ± 2.98 μg/ml and 13.25 ± 4.85 μg/ml, respectively. These results are similar to the BCHA content in food fermented by microbiota [33, 48].

A complex composition of gut microbiota exists in the intestinal environment. The metabolites produced by diverse gut microbiota accumulate in the intestine and show activities through synergistic effects [50,51]. We observed that various strains of gut microbiota produce HIVA and HICA in vitro. These metabolites produced in the complex intestinal environment can accumulate and concentrate in the intestine, potentially exhibiting activities in vivo.

In a mouse model study, the composition of the gut microbiome changed when yogurt containing a concentration of BCHAs about 30 μg/ml was consumed [33]. The BCHAs content of yogurt was similar to the production of them by gut microbiota we observed. Therefore, we inferred that HIVA and HICA produced by gut microbiota may contributes to modulate the gut microbiome in the intestinal environment. These BCHAs are the end products of BCAAs and are produced through the reduction of branched chain keto acids (BCKAs) to BCHA after the transamination of BCAAs to BCKAs [27]. Hydroxyisocaproate dehydrogenase (HicD) enzymes are involved in the reduction of BCKA to BCHA and the expression level of HicD in LAB have been reported to affect BCHA production[48]. The expression of HicD by *Lactobacillaceae* may be associated with their high production of HIVA and HICA compared to other bacterial family group. These relatively high production level suggests that BCHAs can be used as a marker metabolites for *Lactobacillaceae*.

Most gut bacterial species have been grown in complex media of unknown chemical composition, and only a few species have been described in defined or minimal media [52]. In the intestinal environment, there are interaction between microorganisms and there are differences in nutritional condition required for each gut microbiota. Therefore, there are limits to completely mimic the intestinal environment. Because microbial metabolites production may differ depending on environmental conditions, further study is necessary to investigate the BCHAs production under additional culture conditions or in vivo analysis.

Furthermore, we investigated the correlation between bacterial growth variation by HIVA and HICA and their production by gut microbiota. When comparing the two metabolites, HIVA showed a higher and more significant correlation coefficient (r = 0.638, *p* = 0.001). In the case of HIVA, *Lactobacillaceae*, *Lachnosporaceae*, and *Bifidobacteriaceae*, whose growth increased by HIVA treatment, produced relatively high levels of HIVA, showing a similar pattern between the growth modulation and the production. However, in the case of HICA, the growth of *Bifidobacteriaceae* was increased by HICA treatment, but their production level of HICA was not higher than other bacteria. Therefore, HIVA may show a higher correlation coefficient than that of HICA. However, the mechanism of growth modulation or the genetical differences related to HIVA and HICA production are not well understood. Therefore, further studies are needed to explain the differences between two metabolites.

In this study, the effects of HIVA and HICA on the growth of the gut microbiota were evaluated through in vitro screening. HIVA and HICA exhibited growth promoting effects on *Lactobacillaceae* and *Bifidobacteriaceae*; however, HIVA inhibited the growth of *Clostridiaceae* and *Enterobacteriaceae*. They exhibited growth promoting effects on probiotic *Lactobacillaceae* strains and antibacterial effects against *B. fragilis*. These effects may influence the intestinal environment by modulating the composition of the gut microbiome. Additionally, we quantified the production levels of HIVA and HICA by the gut microbiota, which showed family-specific differences. We observed a higher production of these compounds by *Lactobacillaceae*, which could be considered specific marker compounds. Additionally, the bacterial growth variation by HIVA and HICA and production level of them were positively correlated. In this study, we provide basic evidence for the interaction between BCHAs and gut microbiota. Furthermore, this suggests that microbiota-derived BCHAs as active metabolites can potentially contribute to the alleviation of diseases associated with gut microbiota dysbiosis.
